# NanoMAX: the hard X-ray nanoprobe beamline at the MAX IV Laboratory

**DOI:** 10.1107/S1600577521008213

**Published:** 2021-10-05

**Authors:** Ulf Johansson, Dina Carbone, Sebastian Kalbfleisch, Alexander Björling, Maik Kahnt, Simone Sala, Tomas Stankevic, Marianne Liebi, Angel Rodriguez Fernandez, Björn Bring, David Paterson, Karina Thånell, Paul Bell, David Erb, Clemens Weninger, Zdenek Matej, Linus Roslund, Karl Åhnberg, Brian Norsk Jensen, Hamed Tarawneh, Anders Mikkelsen, Ulrich Vogt

**Affiliations:** a MAX IV Laboratory, Lund University, PO Box 118, S-221 00 Lund, Sweden; b Australian Synchrotron, ANSTO, 800 Blackburn Road, Clayton, Victoria 3168, Australia; c Lund University, Synchrotron Radiation Research, 22100 Lund, Sweden; d KTH Royal Institute of Technology, Department of Applied Physics, Biomedical and X-ray Physics, Albanova University Center, 106 91 Stockholm, Sweden

**Keywords:** hard X-ray nanoprobes, coherent diffractive imaging, scanning X-ray microscopy, scanning X-ray diffraction

## Abstract

NanoMAX is the first hard X-ray nanoprobe beamline at the MAX IV laboratory. It utilizes the unique properties of the world’s first operational multi-bend achromat storage ring to provide an intense and coherent focused beam for experiments with several methods. In this paper we present the beamline optics design in detail, show the performance figures, and give an overview of the surrounding infrastructure and the operational diffraction endstation.

## Abstract 2

This work is an overview of the multi-modal hard X-ray nanoprobe beamline at the MAX IV Laboratory. The beamline provides an intense and coherent beam, focused to 40–200 nm in the energy range 5–28 eV.

## 1. Introduction

Scanning hard X-ray microscopy (SXM) offers methods for structure, morphology and composition studies of heterogeneous sample systems. SXM is utilized in material science, life science, cultural heritage, environmental science, nano-technology and archaeology (Mino *et al.*, 2018 ▸; Hémonnot & Köster, 2017 ▸; Cotte *et al.*, 2018 ▸). High spatial resolution and large sample penetration depth allow for detailed studies of volume samples, often in their close to natural state. Instrument and method development is rapid and most low-emittance synchrotron facilities are today operating or developing one or several hard X-ray nanoprobe beamlines (Leake *et al.*, 2019 ▸; Holler *et al.*, 2018 ▸; Chang *et al.*, 2013 ▸; Chen *et al.*, 2014 ▸; Martínez-Criado *et al.*, 2016 ▸; Nazaretski *et al.*, 2017 ▸; Quinn *et al.*, 2021 ▸; Schropp *et al.*, 2020 ▸; Somogyi *et al.*, 2015 ▸; Tolentino *et al.*, 2017 ▸; Winarski *et al.*, 2012 ▸; de Jonge *et al.*, 2014 ▸). A number of technology developments have been important to make SXM attractive to a broader community. Advances in nano-focusing optics fabrication allow focusing to the diffraction limit. Focus sizes often reach below 100 nm with examples below 10 nm (Bajt *et al.*, 2018 ▸). All optics, diffractive, refractive or reflective, have their advantages and disadvantages and no type is best for all energies, focus sizes, working distances and photon flux needs. The development of ultra low-emittance storage rings, first with MAX IV (Tavares *et al.*, 2014 ▸) and soon after ESRF-EBS (Biasci *et al.*, 2014 ▸) and Sirius (Liu *et al.*, 2014 ▸), brings a dramatic increase in coherent flux. The gain in coherent flux is utilized in coherent imaging methods with faster acquisition, improved sensitivity, or to achieve smaller and more intense diffraction-limited foci. On the detector side, the development is also rapid. Hard X-ray pixel detector performance, such as peak intensity, frame rate and dynamic range, has increased with the most modern photon counting and charge integrating detectors (Ballabriga *et al.*, 2011 ▸; Dinapoli *et al.*, 2013 ▸). X-ray fluorescence detectors can handle higher count rates with the latest amplifier and pulse processor technologies (Bordessoule *et al.*, 2019 ▸).

NanoMAX is a hard X-ray nanoprobe beamline at MAX IV, designed to accommodate multiple imaging and scattering methods. The methods are either based on coherence to achieve spatial resolution, such as in ptychography or coherent diffraction imaging (CDI), or on the focused beam providing spatial resolution, such as in scanning diffraction or X-ray fluorescence (XRF) mapping experiments. The beamline will have two endstations to allow for diverse experimental requirements regarding sample environment, energy range, resolution or detector configuration. The diffraction endstation, brought into operation in 2017, exploits the intense coherent photon flux, uses Kirkpatrick–Baez (KB) mirrors for focusing to 40–200 nm and is designed to allow for bespoke sample environments and detector configurations. The main categories of methods regularly used are CDI in forward and Bragg geometries, nano-diffraction in both geometries, and 2D XRF and X-ray absorption near-edge structure spectroscopy (XANES) imaging. Examples of experiments performed are diffraction and strain mapping of nano-wires (Chayanun *et al.*, 2019 ▸; Hammarberg *et al.*, 2020 ▸; Dzhigaev *et al.*, 2020 ▸; Marçal *et al.*, 2020 ▸); single nano-particle coherent Bragg imaging (Björling *et al.*, 2019 ▸, 2020*b* ▸; Dzhigaev *et al.*, 2021 ▸); extreme pressure nano-diffraction (Ji *et al.*, 2020 ▸); ptychographic tomography (Kahnt *et al.*, 2020 ▸); 2D XRF imaging of plant, animal and human cells (Silva Barreto *et al.*, 2020 ▸); nano-diffraction (Nissilä *et al.*, 2021 ▸); and X-ray technology development (Akan *et al.*, 2020 ▸; Chayanun *et al.*, 2020 ▸). The tomography endstation, based on Fresnel zone plate (FZP) optics, is currently under development. It will be optimized to provide 10–50 nm spatial resolution for 2D and 3D tomographic experiments with XRF, XANES contrast and CDI as the primary imaging methods. The microscope will operate under vacuum and feature liquid nitrogen cooling to mitigate radiation damage in sensitive samples. The beamline has several detectors that can be shared between the two endstations and a configurable control system to allow integration of user equipment (Björling *et al.*, 2021 ▸).

**Figure 1:**
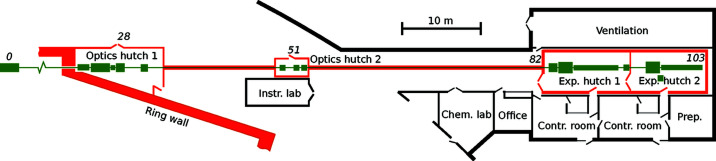
NanoMAX floor plan. The beamline is shown in green in the main and satellite buildings. Red indicates radiation-controlled hutches and the shielded beam-transportation tube. Approximate distances in metres from the undulator are shown in italics.

NanoMAX is located in sector three of the MAX IV 20-fold symmetric, 3 GeV, 528 m circumference storage ring. The beamline area is shown in Fig. 1 ▸ and main storage ring and beamline parameters are listed in Table 1 ▸. The beamline is 103 m long and extends out of the main experimental hall into a satellite building.

### 1.1. Design criteria

The realized beamline is the result of a number of design criteria established early in the project phase, through input from various stakeholders as well as through practical constraints as follows: the beamline was built simultaneously with six other beamlines (Klementiev *et al.*, 2016 ▸; Enquist *et al.*, 2018 ▸; Ursby *et al.*, 2020 ▸; Zhu *et al.*, 2021 ▸) and the two storage rings, meaning technical design resources were limited and commercial vendors preferred. The optics scheme should only be horizontally deflecting, beneficial for beam stability and have few optical elements to preserve the brilliance. It should be possible to equip the beamline with new nano-focusing elements in the future hence a secondary source was deemed necessary. Based on the wide-spread user interest the energy range should span a large range to allow excitation of elements, for example, used in nano-technology or found in environmental science. The diverse experimental demands warranted two endstations, where the KB-focusing enables a large energy range and free working distance, while the Fresnel zone plates can provide higher resolution, but over a more limited photon energy range and with limited space around the sample.

## 2. Undulator and front-end

**Figure 2:**
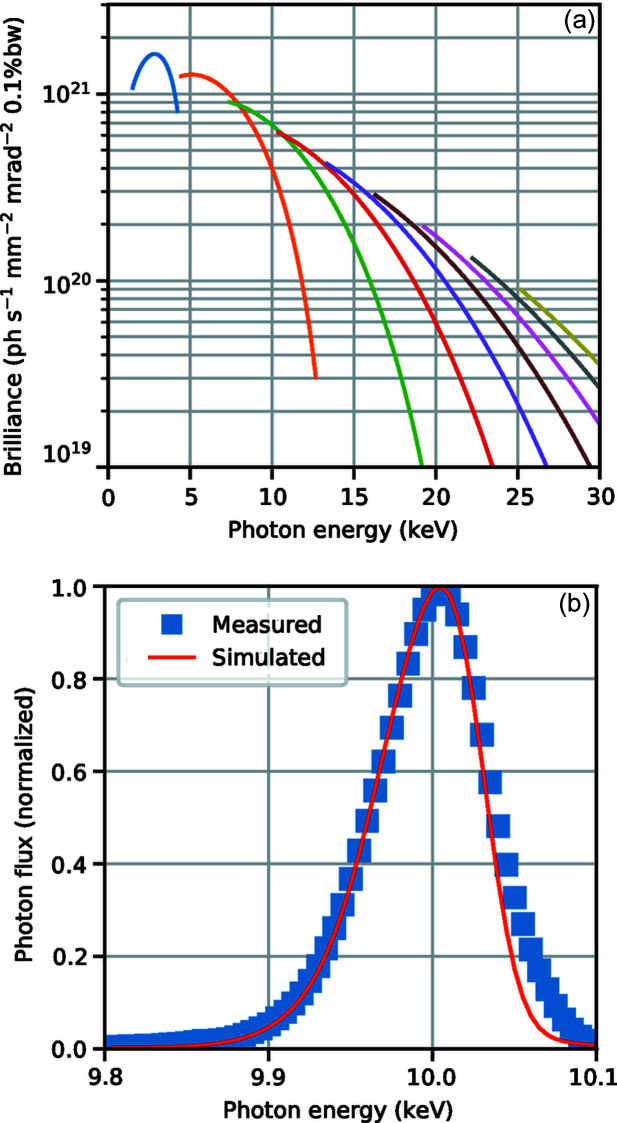
(*a*) Calculated brilliance for the odd harmonics 1–17 at 500 mA for *K* between 0.5 and 2.10. Simulations were carried out in *SPECTRA* (version 10.2; Tanaka & Kitamura, 2001 ▸) with Gaussian approximation and storage ring parameters from Table 1 ▸. (*b*) Fifth-harmonic undulator peak measured in the first experimental hutch at 5.2 mm undulator gap and at 250 mA ring current with a corresponding simulated spectrum.

The undulator at NanoMAX (Hitachi Metals, Japan) is designed to be a brilliant photon source in the energy range 5–28 keV. It is an in-vacuum, room-temperature, permanent magnet, hybrid design (Yamamoto *et al.*, 1992 ▸) with a maximum *K*-value of 2.10 (Attwood & Sakdinawat, 2017 ▸). The main undulator parameters are summarized in Table 2 ▸. The physical length of the undulator is 2.8 m, although the length of the straight section is 4 m. The choice for a shorter undulator was made in order to not challenge the initial operation of the ring and for easier heat load management. Fig. 2 ▸ shows the calculated brilliance for the odd harmonics (*a*). The first harmonic is outside of the energy range of the beamline. A measurement of the fifth harmonic peak at 10 keV together with a simulation of the same peak is shown in (*b*). The undulator can be tapered in order to broaden peaks (Tarawneh *et al.*, 2019 ▸) at the cost of photon flux when, for example, energy scanning experiments are performed.

**Figure 3:**
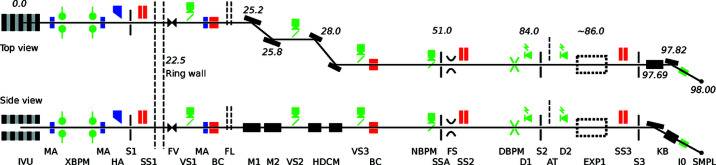
Top and side views of the beamline optics together with most components for diagnostics and beam conditioning along the beamline. Approximate distances in metres from the undulator are shown adjacent to the main components. IVU: in-vacuum undulator; MA: heat-absorbing masks; XBPM: X-ray blade beam-position monitor (BPM); HA: actuated heat absorber; S1: L-shaped movable masks; SS1–3: radiation safety shutters; FV: fast-closing valve; VS1–3: fluorescence view screens; BC: bremsstrahlung collimators; FL: diamond heat filters; M1: vertically focusing mirror; M2: horizontally focusing mirror; HDCM: horizontal double-crystal monochromator; NPBM: high-resolution BPM; SSA: secondary source aperture; FS: fast shutter; DBPM: diamond-BPM; D1–2: pin-diodes; S2: slits; AT: multiple attenuators; EXP1: place for tomography endstation; S3: KB-slits; KB: nano-focusing mirrors; I0: miniature ion-chamber; SMPL: sample position.

The undulator can generate a maximum of 6.5 kW in total power at 500 mA stored beam, which is exposed to the front-end [FMB Berlin, Germany; Bartalesi *et al.* (2016 ▸)]. The front-end consists of three fixed heat-absorbing masks, two blade-type X-ray beam position monitors, two movable L-shaped masks to define beam divergence angle, a heat absorber, a tandem safety shutter, a low power view screen, an electron beam deflector, a bremsstrahlung collimator and a fast-closing valve activated on vacuum leaks (see Fig. 3 ▸). The front end designs at MAX IV are similar for all (hard X-ray) beamlines and its main functions are setting the acceptance angle and reducing the heat-load of the undulator beam to acceptable levels. At NanoMAX the beam is masked to 100 µrad × 100 µrad and a maximum of 500 W is allowed to pass into the first optics hutch where further heat reduction takes place.

Heat load on the mirrors and the monochromator can deteriorate the focusing performance. A fixed heat mask is therefore located early in the first optics hutch where it reduces the beam acceptance angle further to 50 µrad (H) × 40 µrad (V) and thereby the heat load to a maximum of 100 W. A set of water-cooled diamond filters effectively removes the first undulator harmonic.

## 3. Beamline optics

The beamline optics layout is schematically depicted in Fig. 3 ▸, together with most heat-load (blue), radiation safety (red) and diagnostics components (green). With the request for a large energy range, we decided to use X-ray mirror optics for focusing because they are achromatic. The design where the main mirrors and especially the monochromator deflect the beam in the horizontal direction favours stability. The sensitivity to angular errors in optical components is lower in the horizontal direction because the photon source in the undulator is strongly elliptical in shape (see Table 1 ▸). Horizontally deflecting optics is the chosen design for several hard X-ray nanoprobe beamlines (Chang *et al.*, 2013 ▸; Howard *et al.*, 2020 ▸; Leake *et al.*, 2019 ▸; Li *et al.*, 2017 ▸; Quinn *et al.*, 2021 ▸; Somogyi *et al.*, 2015 ▸) at third- and fourth-generation sources.

### 3.1. Primary mirrors

The first mirror (M1 in Fig. 3 ▸) is a sagittally focusing Pt coated cylinder. The second (M2) is a bendable meridionally focusing mirror with three coating stripes, Pt, Rh and Si, for high-energy photon rejection. The two mirrors (Table 3 ▸, Zeiss, Germany) image the undulator source, approximately 1 to 1, onto the secondary source aperture at 51 m distance. Due to the brilliant undulator source with the narrow photon beam, the mirrors can be relatively short (400 mm optical length) and are arranged close to each other in a common vacuum chamber. Linear motions, in horizontal and vertical directions, of the two mirrors are achieved in stages outside and below the vacuum chamber. All external mechanics are mounted on a common granite block. The use of a common granite and vacuum chamber makes the assembly rigid and compact (FMB Oxford, UK). Bending and rotational motions, in pitch and yaw, are handled inside the vacuum chamber. The different mirror coatings on the second mirror are selected by vertical translation of the mirror. Both mirrors are water-cooled via a cooling blade, acting as a heat exchanger. The blade is inserted in a slit in each mirror and the slit is filled with indium–gallium eutectic. This design provides good thermal conductivity and mechanical separation from vibrations in the cooling water circuitry. A retractable fluorescence view screen (VS2 in Fig. 3 ▸) is available after the mirror chamber to aid in mirror adjustments.

### 3.2. Monochromator

The monochromator (FMB Oxford, UK), a double-crystal Si(111) fixed-exit design with horizontal diffraction geometry (Kristiansen *et al.*, 2016 ▸), is placed at 28 m from the undulator source, right after the two main focusing mirrors. The horizontal diffraction geometry has the drawback of reduced transmission at low energies due to the horizontal polarization of the synchrotron radiation. In comparison with a classic vertically diffracting monochromator the flux reduction is about 50% at 5 keV but disappears above 7 keV. The two monochromator crystal reflections offset the beam by 10 mm, which together with a 3 mm offset from the mirror pair result in 13 mm total beam displacement in the first optics hutch. This offset is sufficient for the Bremsstrahlung collimators needed to capture gamma radiation from the storage ring straight section, while allowing the synchrotron beam to pass. The small monochromator offset allows the two crystals to be short, 50 and 90 mm, and the crystal cage design can be made compact and rigid. The first crystal is mounted on the Bragg goniometer without any further motorized adjustments. The second crystal has motor and piezo-actuator adjustments for pitch and roll. The crystals are liquid nitrogen (LN2) cooled where the first crystal is side-cooled through copper blocks with direct circulating liquid nitrogen from a cryo-cooler. The second crystal is cooled via braids from the first crystal. The direct cooling of the first crystal is needed because the main part of the remaining heat load is absorbed here. For the second crystal, braid cooling is sufficient since the heat load is considerably lower. However, it is important that the two crystals have the same temperature, thereby the same crystal lattice constant, to give the same Bragg reflection condition. With the first rigid crystal mounting, vibrations are kept at the required specification (Kristiansen *et al.*, 2016 ▸). The second crystal, with its adjustments, is more susceptible to vibrations but the arrangement with braid cooling limits vibration transfer. A retractable fluorescence view screen (VS3 in Fig. 3 ▸) is available after the monochromator for viewing the beam during monochromator adjustments. All screens along the beamline were used during initial beamline commissioning but are rarely needed today.

For photon-hungry experiments, a double multilayer monochromator (DMM) can be advantageous with its lower energy resolution (Δ*E*/*E* ≃ 10^−2^) leading to substantially higher photon flux. The beamline is prepared for an upgrade with space for a DMM downstream of the existing DCM. The beamline would then use one of the monochromators while the other is bypassed. It should be noted, however, that the lower energy resolution may not be compatible with the use of high-resolution diffractive optics.

### 3.3. The secondary source

KB-optics do not require a stigmatic source since the focusing is independent in the vertical and horizontal directions. However, high-resolution zone plates require a symmetric wavefront curvature for best performance and therefore the beamline uses a stigmatic secondary source. The beamline is then also well prepared for future developments in nano-focusing optics. The secondary source size is chosen to match the coherence length to the acceptance aperture of the KB-optics or FZP diameter when diffraction-limited focusing is needed, or when the experimental method requires coherent illumination. Larger openings are used, when a larger focus can be accepted, to gain in photon flux. For example, in XRF mapping, the highest resolution is not always requested, and coherence is not important.

The secondary source aperture (SSA, FMB Oxford, UK) is located 51 m from the undulator source in a small optical hutch (Figs. 1 ▸ and 3 ▸). The aperture size is defined with high-precision vertical and horizontal slit blades, guided by flexure-based precision mechanics.

**Figure 4:**
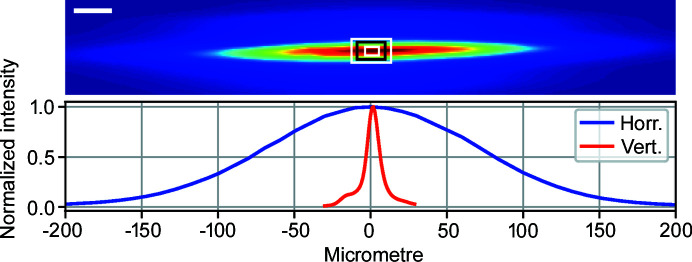
Monochromatic X-ray beam profile on the secondary source aperture measured at 14 keV. Secondary source aperture sizes for different operation modes are indicated in the intensity profile. From small to large aperture: full coherence illumination, high flux illumination and 100 nm spot size at the KB-focus. The scale bar is 25 µm. Intensity profiles in the vertical and horizontal directions show a FWHM of 160 µm (H) × 10 µm (V).

Fig. 4 ▸ shows an image of the undulator source at the secondary source position measured at 14 keV, together with horizontal and vertical intensity profiles. The intensity profile was acquired by raster scanning the SSA position in the beam profile with the aperture set to 5 µm (H) × 1 µm (V), while recording the signal on a diode. Three SSA apertures are depicted to illustrate different operation modes. The smallest aperture corresponds to coherent illumination of the KB-optics; the middle aperture to high flux mode; and the largest aperture results in partially coherent, high photon flux illumination and 100 nm focus size. The measured intensity profile size in the vertical direction is in agreement with ray-tracing and heat-load simulations. In the horizontal direction the measured size (160 µm) differs from the simulated value (130 µm) due to a mechanical limitation in the mirror bending. The issue will be corrected during the next mirror chamber intervention.

Stability of the photon beam in position, angle and intensity is key to preserve the brilliance of the MAX IV 3 GeV storage ring. Beamline components are designed to have eigenfrequences above 55 Hz to achieve long-term stability and low vibration amplitudes. However, it is inevitable that with changing heat-load and with time, the mirrors and the monochromator crystals will drift in angles, leading to mis­alignment of the photon beam on the secondary source aperture. A nano-beam position monitor (NBPM, FMB Oxford, UK) is located upstream of the SSA (see Fig. 3 ▸) to monitor the position and intensity of the beam, incident on the SSA. The position values are used to control the second crystal of the monochromator in its pitch and roll angles with piezo actuators, in closed-loop operation. In this way the photon beam is always steered to point through the SSA. It should be noted that closed-loop operation can only compensate for small drifts, and beam steering therefore relies on static stability of the two mirrors and the first crystal of the monochromator. The NBPM is normally operated at 10 Hz. Our experience is that this arrangement is reliable.

The photon beam after the secondary source is transported in a radiation shielded vacuum tube into the first experimental hutch, where a chamber with diagnostics and beam conditioning components is placed. This chamber hosts a diamond beam-position monitor [DBPM, Cividec, Austria; Griesmayer *et al.* (2019 ▸)], slits, diodes, attenuators and a motorized phase plate, as shown in Fig. 3 ▸. The DBPM measures the beam position with sub-micrometre resolution at kilohertz frequency and is currently in commissioning. The aim is to integrate it into the regular data acquisition where it can be used to validate data quality. The slits are used to adapt the beam divergence to the downstream nano-focusing optics. The phase plate is used to generate elliptically polarized light useful for studies of, for example, magnetic sample systems. The present phase plate operates in the 7 keV regime, but can be exchanged with other phase plates if requested.

## 4. The diffraction endstation

The diffraction endstation (Johansson *et al.*, 2018 ▸) is designed with emphasis on diffraction and scattering experiments using bespoke sample setups. It is located in the second experimental hutch, 47 m from the SSA (see Fig. 1 ▸). The location in the second hutch has the practical advantage that experimental preparations can be performed while experiments are running in the first hutch. Here, we provide a general description of the endstation together with details about the KB-optics and the focusing performance. The technical design of the diffraction endstation, its different experimental methods and performance figures will be presented in detail in a separate article (Carbone *et al.*, 2021 ▸).

**Figure 5:**
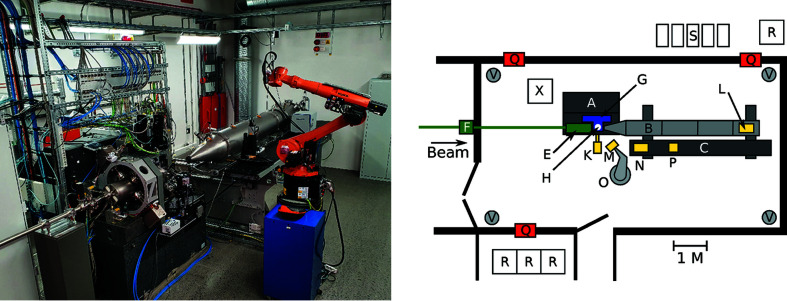
Left: photograph of the diffraction endstation with the detector robot and the vacuum flight tube. Right: top view of the diffraction endstation hutch. A: granite support, B: detector vacuum flight tube, C: breadboard for detectors and equipment, E: KB-optics chamber, F: radiation safety shutter, G: goniometer, H: sample position, K: fluorescence detector, L: Eiger detector, M: Merlin detector, N: Pilatus detector, O: detector robot, P: Crytur detector, Q: chicanes, R: electronics cabinets, S: pumps, chillers *etc.* V: ventilation exhaust. Ventilation inlet is through the hutch ceiling (not shown).

The endstation is designed around the KB-optics and a two-circle sample goniometer (see Fig. 5 ▸). The mirror chamber and the goniometer are supported on a 7 ton granite block which is grouted to the floor. Experiments in the Bragg geometry require a photon-counting pixel detector placed at an off-axis angle. Instead of using a detector arm which rotates around the sample position, common on diffraction endstations, we use an industrial robot (Cybertech KR20 R1810, Kuka, Germany) for positioning the detector (Merlin 250k, Quantum Detectors, UK) at the Bragg peak of interest. The detector robot is programmed to be positioned by applying polar coordinates with sample and X-ray focus position as the origin. A vacuum flight tube downstream of the sample houses a photon-counting pixel detector (Eiger2X 4M, Dectris, Switzerland). The forward sample-to-detector distance can be varied from 1.0 to 4.5 m by adding or removing sections in the flight tube. An optical breadboard beside the flight tube can be moved into the forward direction to support temporary detector setups, while parking the flight tube to the side. For example, the beamline scintillator X-ray camera (CRYCAM, Crytur, Czechia), the pixel detector (Pilatus 2 1M, Dectris, Switzerland) used for wide-angle X-ray scattering (WAXS) measurements or a user-supplied detector can be mounted here. An XRF-detector (Sirius-SD one-element, RaySpec, UK), with a high-performance pulse processor (Xspress3, Quantum Detectors, UK) is available for XRF experiments. The XRF detector is normally used in air, in the horizontal plane, at a 60–90° angle to the incident X-ray beam.

Two optical microscopes are used to navigate the sample to the measurement position. One microscope views the sample in the forward beam direction. The other views the sample from above and is used in centre-of-rotation alignment, and in diffraction and tomography experiments. The free distance between the mirror chamber exit window and the sample position is 115 mm but part of the distance is occupied by a mirror for the optical microscope, a clean-up aperture and a small ion chamber monitoring the beam intensity. The practical free distance for custom sample environments is approximately 50 mm. However, by moving the above-mentioned components out, larger environments can be fitted.

**Figure 6:**
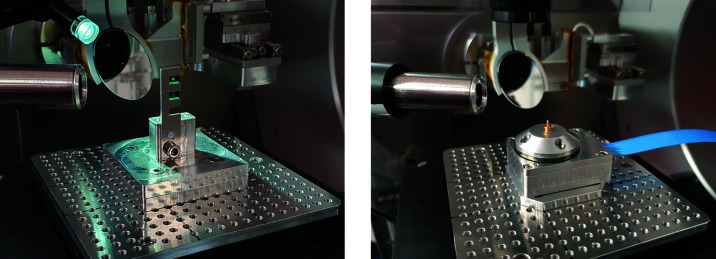
Images of samples mounted at the focal position. Left: two silicon nitride windows with biological samples mounted on a standard sample holder. Right: miniature rotary stage used for tomography measurements. The sample is mounted on a point-shaped sample pin.

We give two examples in Fig. 6 ▸ of how samples are typically mounted on the sample scanner. For 2D-mapping of biological samples on silicon nitride windows, a simple aluminium stick is used. Up to three samples can be mounted on the stick, making batch scanning possible. A point-shaped sample pin is sometimes needed, *e.g.* in tomography experiments. We have adopted a pin design developed by Holler *et al.* (2017 ▸) as a sample mount standard. Other customized sample mounts are often created according to the needs of a specific experiment.

### 4.1. Kirkpatrick–Baez optics

The diffraction endstation uses total reflection, fixed curvature, X-ray optics in a KB arrangement, with quality specifications enabling diffraction-limited focusing (JTEC, Japan, see Table 4 ▸). A diffraction-limited focus is defined by the wavelength of the X-rays and the numerical aperture (NA) for the focusing mirrors. The FWHM of the KB-mirror focus is 

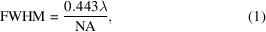

where NA is defined by the half-angle Θ of the light focused by the optics and the refractive index *n*, which is unity for mirrors, 

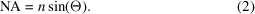

For our KB mirrors, the NAs in the vertical and horizontal directions are 6.1 × 10^−4^ and 6.2 × 10^−4^, respectively, resulting in similar resolutions in both directions. Note that the NA for hard X-ray optics is several orders smaller compared with visible light microscopy where it often is close to 1. Therefore, the resolution and wavelength can be similar for visible light but this is far from the case with X-rays. An advantage of a small NA for X-rays optics is that the depth of focus (DOF) is very large compared with the resolution. DOF is defined as 

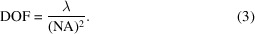

The KB-optics have 120 µm (28 keV) to 650 µm (5 keV) DOF which, in combination with the large X-ray penetration depth, allows measurements of samples considerably thicker than the transverse resolution.

The parameters of the KB mirrors are shown in Table 4 ▸. The broad energy range, 5–28 keV, dictates which reflective coating as well as the possible beam incidence angles on the two mirrors that can be specified. The mirrors have a Pt coating which affords good reflectivity for most of the energy range, but drops rapidly above 25 keV for the chosen incidence angles. Pt also has *L*-absorption lines between 11.5 and 13.9 keV causing reflectivity drops. Large demagnification is needed to achieve the desired focus, which leads to strongly elliptical mirror shapes. This means that the incidence angle varies (2.2–3.0 mrad) along both mirrors, which in turn results in varying reflectivity at higher energies. The lengths of the mirrors are optimized to give similar NAs in the vertical and horizontal directions, and thereby a symmetric focal spot.

**Figure 7:**
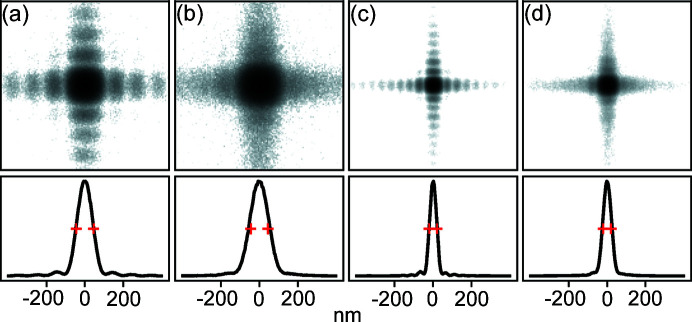
KB optics. Simulation of focusing properties using mirror parameters from Table 4 ▸ and measured mirror figure errors. Top row: (*a*, *b*) simulations of the focus at the sample position for 10 keV, (*c*, *d*) for 22 keV. (*a*, *c*) SSA is set to coherence mode. (*b*, *d*) SSA is set to flux mode. Intensities in all images are on normalized logarithmic scales to visualize the diffraction pattern. Bottom row: intensity profiles on normalized linear scales for the corresponding upper image. Red marks indicate the ideal resolution according to equation (1).

**Figure 8:**
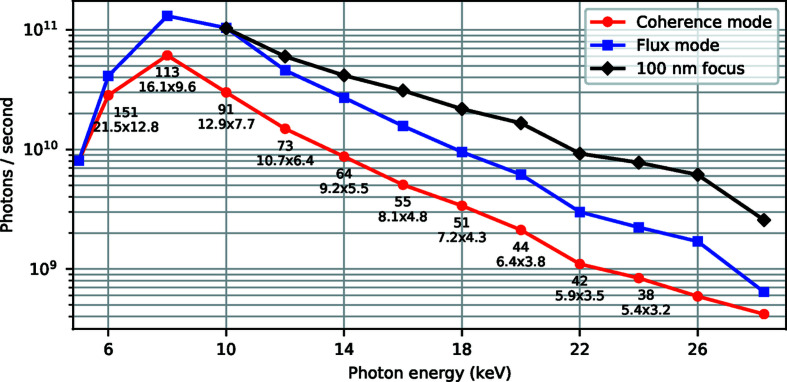
Photon flux measured at the sample position with a PIN diode for different SSA settings and energies at 300 mA ring current. Red curve: flux for highest degree of coherence. Blue curve: flux at partial degree of coherence and with 20% larger focus size than for high coherence. Black curve: flux for SSA opening resulting in 100 nm spot size for energies above 10 keV. Measured focus sizes (nm) and used SSA openings [H × V (µm)] are shown below the coherence mode flux markers.

The mirror quality was measured by the manufacturer to have figure errors below 1 nm peak-to-valley and micro roughness below 0.15 nm r.m.s. The excellent mirror quality is essential to reach diffraction-limited focusing for the whole photon energy range. The mirror focusing characteristic has been simulated, taking the mirror errors and diffraction effects into account, using the *Oasys* synchrotron software suite (Rebuffi & Sánchez del Río, 2017 ▸) with the *ShadowOui* extension (Rebuffi & Sánchez del Río, 2016 ▸). Fig. 7 ▸ shows the resulting focus profile for four cases. The wavelength-limited focusing is clearly visible where (*a*) and (*b*) show larger focus at 10 keV compared with (*c*) and (*d*) for 22 keV, in accordance with equation (1). In plots (*a*) and (*c*) the SSA was set to give fully coherent illumination (see Table 5 ▸) governed by the Van Cittert–Zernike theorem (Attwood & Sakdinawat, 2017 ▸; Björling *et al.*, 2020*a* ▸). Plots (*b*) and (*c*) show the focus with the SSA more open, which gives a partially coherent illumination and results in a 20% larger spot size. We call the two modes of illumination ‘coherent mode’ and ‘flux mode’, respectively. The flux mode SSA setting is two times larger than the corresponding coherent mode SSA opening, in both the vertical and horizontal directions, resulting in 3–4 times more intense beam (see Fig. 8 ▸). This mode of operation is useful in, for example, XRF or X-ray diffraction (XRD) experiments where coherent illumination is not needed.

Fig. 8 ▸ shows photon flux curves measured with a PIN diode at the sample position, for different SSA settings and energies. Below the coherence mode curve are values for measured focus sizes and SSA openings shown. The focus sizes have been determined by ptychographic measurements of a test structure followed by ptychographic reconstruction and numerical wave propagation, described in detail by Björling *et al.* (2020*a* ▸).

The two KB mirrors need to be aligned accurately to obtain a stigmatic focus. The most sensitive adjustments are the pitch angles of the mirrors. These must be controlled to 20 nrad accuracy while the roll and yaw angles only need 10 and 100 µrad accuracy, respectively. Pitch angles for both mirrors and roll for one mirror are adjusted by piezo actuators inside the vacuum chamber. Transverse and longitudinal mirror positions were adjusted during installation.

**Figure 9:**
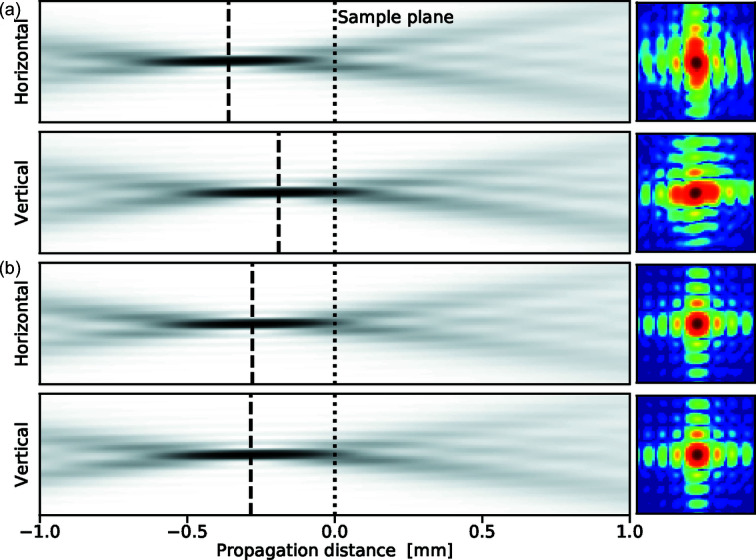
Longitudinal beam profiles from ptychographic measurements of a test structure, followed by ptychographic reconstruction and numerical propagation. The two upper plots (*a*), with horizontal and vertical profiles, show an astigmatic focus. After adjustment of the KB-mirror pitches, the focus becomes stigmatic as shown in the two lower plots (*b*). The right-hand side of every plot shows intensity images on logarithmic scale for the indicated best horizontal, respective vertical, focus positions.

The KB mirror pitches need to be fine-tuned regularly. Typically the mirrors are checked and, if needed, adjusted daily or weekly in a convenient procedure using ptychographic imaging (Faulkner & Rodenburg, 2004 ▸; Rodenburg & Faulkner, 2004 ▸; Thibault *et al.*, 2008 ▸) of a test pattern. The steps of the process are (i) to collect a small ptychographic scan from the test pattern; (ii) to reconstruct the complex probe and object (Thibault *et al.*, 2009 ▸); (iii) to numerically propagate the recovered wavefront along the beam (Enders & Thibault, 2016 ▸), identifying the focal positions; and (iv) to adjust the mirror pitches. This procedure is repeated a few times, which typically takes in total 10 to 15 min. Fig. 9 ▸ shows an example of the propagated beam before and after the adjustment procedure.

Spatial resolution and focus size can be measured directly, for example, with knife-edge scans, imaging of periodic test patterns revealing contrast, or indirectly by ptychography and probe reconstruction. It is good to recall the difference between the terms ‘spatial resolution’ and ‘focus size’. The focus size is only one part of the resolution. Obtainable spatial resolution for scanning nanoprobes is a combination of beam focus, contrast of the sample, positioning accuracy and statistical noise in data acquisition. Resolution can be improved, for example, by acquiring data for a longer period of time or by eliminating air scattering in the beam path while the focus size remains constant.

**Figure 10:**
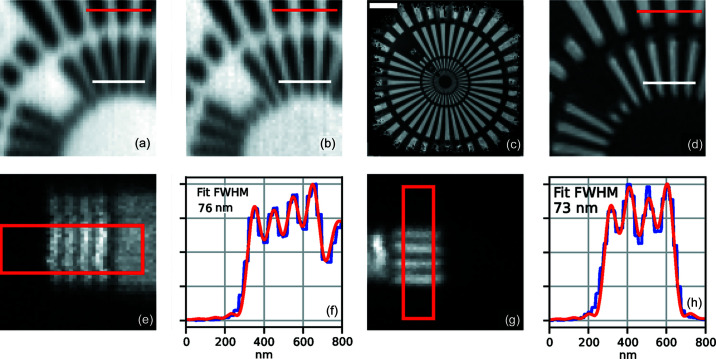
(*a*)–(*d*) Direct and ptychographic spatial resolution measurements of a Siemens star pattern in tantalum. (*a*) Total yield fluorescence emission of the 50 nm central part, measured at 10 keV excitation energy. (*b*) Same as A but measured at 22 keV. (*c*) Ptychography reconstructed phase image of the Siemens star measured at 10 keV. (*d*) Close-up of the 50 nm features in (*c*). Scalebar in (*c*) is 2 µm. (*e*)–(*h*) Direct spatial resolution measurement of periodic chromium line patterns on a silicon nitride window with 50/50 nm wide lines/separations measured at 16 keV in XRF. (*f*) and (*h*) Line profiles (blue) integrated from the rectangles in (*e*) and (*g*), respectively, and the simulated profiles (red).

In Figs. 10 ▸(*a*)–10(*d*), a Siemens star test chart (XRESO-50, NTT-AT, Japan) was measured in total yield fluorescence at 10 keV (*a*) and 22 keV (*b*), and with ptychography at 10 keV in coherence mode (*c*) and (*d*). Contrast was determined along the red and white lines with 200 nm and 100 nm wide line/space periods, respectively. In (*a*) and (*b*), where the X-ray focus size, to a large extent, determines the resolution, the contrast is 34% and 9% (red/white) at 10 keV (*a*) and 50% and 21% (red/white) at 22 keV (*b*). By defining the resolution limit as when the contrast vanishes below 10%, the contrast on the 100 nm line/space periods in (*a*) corresponds well with the predicted focus size of 93 nm (see Table 5 ▸). To determine the resolution limit at 22 keV by measurement, a smaller test structure would have been needed. However, by extrapolating from the measured contrast values to 10% contrast, the limit is estimated to be 60 nm, which should be compared with the simulated 43 nm. In (*c*) and (*d*), the contrast is 100% and 88% (red/white) and the finest spokes are clearly visible because, in ptychography, the resolution is not limited by the focused beam size but by the scattering angle, shot noise, scanning accuracy and instrument stability.

Figs. 10 ▸(*e*) and 10(*g*) show a set of 50 nm-wide chromium lines with 50 nm separation on a silicon nitride window measured by X-ray fluorescence at 16 keV photon energy. Figs. 10(*f*) and 10(*h*) show line profiles (blue) from the rectangles in (*e*) and (*g*), together with profiles (red) generated by convolving the known periodic test structure and a beam profile according to Björling *et al.* (2020*a* ▸). The convolved profile is fitted to determine the FWHM width of the beam focus. The resulting widths, 76 and 73 nm, are larger than the focus size of 55 nm at 16 keV measured by ptychography.

The results from the direct measurements on the Siemens star and Cr test patterns show focus sizes larger than the corresponding sizes determined by ptychography (see Fig 8 ▸). The discrepancy is likely the result of imperfect sample scanning and position recording in the measurements. Ptychographic algorithms can partly compensate for position errors whereas the errors directly contribute to the measured focus size in the direct measurements. Additional beam property investigations have been published in earlier work by Osterhoff *et al.* (2019 ▸), Björling *et al.* (2020*a* ▸) and Chayanun *et al.* (2020 ▸).

## 5. Infrastructure

**Figure 11:**
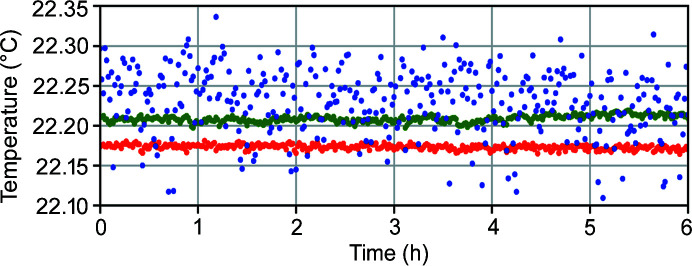
Temperature measured in the diffraction endstation hutch under static conditions for 6 h. Temperature of the sample (blue), the KB-vacuum chamber (green) and the granite (red). The temperature of the sample fluctuates more than for heavier components. 2σ deviations for respective temperature are 83 mK (sample), 9 mK (chamber) and 8 mK (granite).

The beamline infrastructure, such as the floor, hutches, ventilation and water system, is designed to minimize mechanical vibration, acoustic and cultural noise, and thermal drift. To this end, the first optics hutch is constructed from steel and lead, and temperature-controlled to better than 0.5 K with recirculating ventilation. The two experimental hutches are built from concrete with a thermal insulation sandwich, where air between the hutch walls and roof towards the surrounding rooms supports thermal stability. Each experimental hutch has a recirculating ventilation system with large area air inlets in the ceiling and air outlets at the floor level. This design aims at providing a slow, laminar downward air flow resulting in thermal stability within 0.1 K at the sample position over weeks. Fig. 11 ▸ shows temperatures measured at three positions in the vicinity of the sample over 6 h during which the experimental hutch was closed. The sample temperature varies most due to the low mass of the used aluminium sample stick while the KB optics chamber and the granite support are stable to within a few mK. All temperature fluctuations are, however, well below the design goal of 0.1 K. Upon entering the hutch, it can take up to a few hours to reach stable conditions after closure, depending on the disturbance. To minimize thermal drift from active heat sources, most electronics are located outside of the hutch in designated rooms. Electronics that must remain close to the experiment, for example piezo controllers, electrometers and detector processors, are housed in a ventilated rack close to the experimental setup.

**Figure 12:**
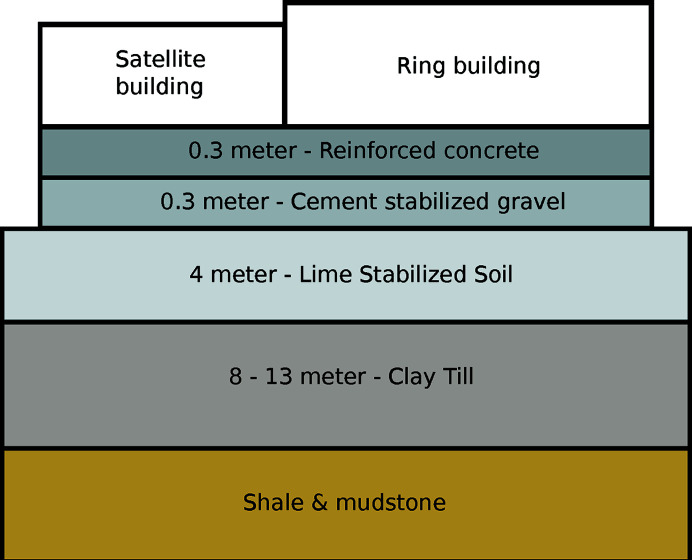
Foundation for the MAX IV main building and the NanoMAX satellite house.

The floor for the 3 GeV storage ring and all its beamlines is cast to constitute one unit (Tavares *et al.*, 2018 ▸). This is to create one foundation with low vibration amplitude, due to its mass, where components close by on the floor are moving coherently. The floor vibration is, on average, below 10 nm r.m.s. and the dominating eigenfrequences are in the range 5–18 Hz. A cross-section of the floor construction is illustrated in Fig. 12 ▸. All optical components are mounted on granite blocks, which are grouted to the floor. Equipment generating vibrations, such as vacuum pumps and chillers, are suspended on springs, and tuned to 2–5 Hz to reduce vibration transmission to the floor. The outdoor area around the satellite building is blocked for traffic to further reduce ground vibrations. Two seismometers are in use at the experimental stations to monitor ambient vibrations, and to detect any detrimental changes in the local environment.

## 6. Beamline operation

### 6.1. Support facilities

A laboratory dedicated to sample preparation for NanoMAX users is located at the end of the satellite building (see Fig. 1 ▸). The laboratory is equipped with a laminar flow bench for clean sample handling, a high-resolution optical microscope (Olympus BX53M), a long-distance stereo microscope (Olympus SZX16), a fume hood for basic chemistry work and a user-friendly scanning electron microscope (Hitachi SU1000). A dedicated chemistry laboratory, shared with other beamlines, is available to users and conveniently located close to NanoMAX. MAX IV has one central biology laboratory which also is available to users after training.

### 6.2. Control system and data handling

The MAX IV Laboratory uses *Tango* (Chaize *et al.*, 1999 ▸) as the general control system for all beamlines, storage rings and the linear accelerator. Most equipment at the beamline is controlled via Tango device servers. A lightweight Python framework named *Contrast* is used for orchestrating the beamline control and data acquisition on top of the Tango devices (Björling *et al.*, 2021 ▸). All high data-rate detectors stream data independently to receiving software for analysis on-the-fly or storage on the MAX IV centralized file storage. A compute cluster at MAX IV is available for data analysis during and shortly after experiments. The cluster is used for tasks like ptychographic reconstructions, azimuth or radial integration of diffraction data, X-ray fluorescence spectral fitting, and general instant data visualization during beam time. The data acquisition and data analysis tools available at NanoMAX are continuously developed by the beamline team, the scientific software group, the detector group and the control system group.

## 7. Conclusions

NanoMAX is a nanoprobe beamline that allows experiments with multiple methods and possesses a unique quality with its intense coherent X-ray beam. We have brought the beamline and the diffraction endstation into user operation with scanning diffraction, coherent diffractive imaging and X-ray fluorescence mapping as our main methods, whilst also providing a platform for exploring other techniques. The performance results of the beamline and the KB optics of the diffraction endstation are in good agreement with the design figures, where near-diffraction-limited focusing is achieved for the full energy range, together with a substantial flux in a fully coherent beam, peaking at 8 keV with 6 × 10^10^ photons s^−1^. The diffraction endstation can accommodate customized experimental environments and is equipped with detectors for Bragg and forward scattering geometries, and a fluorescence detector for elemental mapping. A tomography endstation, currently under development, will complement the diffraction endstation and add more experimental opportunities to the users of NanoMAX.

## Figures and Tables

**Table 1:** MAX IV 3 GeV storage ring and NanoMAX beamline main parameters

Storage ring energy	3 GeV
Nominal design current	500 mA
Current (operation May 2021)	300 mA
Electron beam emittance	326 pm rad (*x*), 8 pm rad (*y*)
Electron energy spread	7.7 × 10^−4^
Electron source size	54 µm (σ_*x*_), 4 µm (σ_*y*_)
Electron source divergence	6 µrad (σ_*x*_), 2 µrad (σ_*y*_)
Insertion device	In-vacuum undulator
Photon energy range	5–28 keV
Beamline optics	Vertical and horizontal focusing with mirrors onto secondary source
	
Monochromator	Cryo-cooled Si(111), double crystal, horizontal diffracting geometry
	
Endstation 1	In-vacuum tomography station with Fresnel zone-plate optics for highest resolution (under development)


Endstation 2	Versatile coherent diffraction station with KB optics (operational)
	
Detectors	Eiger 2X 4M, Merlin Si Quad 512 K, Pilatus 2 1M, RaySpec one-element SDD, Crycam X-ray camera with Andor Zyla 4.2+



**Table 2:** Undulator parameters

Number of periods	111
Period length	18 mm
Maximum *K*-value	2.10
Minimum magnetic gap	4.2 mm
Maximum taper	0.5 mm/2 m
Magnet material	NdFeB
Pole material	Vanadium permendur
Phase error for all gaps (r.m.s.)	<2.0

**Table 3:** Beamline optics parameters

Mirror 1 – vertically focusing	
Mirror shape	Fixed curvature circular cylinder, horizontally deflecting

Sagittal radius	68.9 mm
Incidence angle	2.7 mrad
Slope error	0.5 µrad RMS
Optical length	400 mm
Coating	Pt, 40 nm
Substrate	Si
	
Mirror 2 – horizontally focusing	
Mirror shape	Bendable curvature circular cylinder, horizontally deflecting

Meridional radius	9.44 km (nominal)
Incidence angle	2.7 mrad
Slope error	0.3 µrad RMS
Optical length	400 mm
Coatings	Si, Rh, Pt, 40 nm
Substrate	Si

**Table 4:** KB optics parameters

Mirror	Vertical focusing	Horizontal focusing
Mirror material	Single crystal silicon	Single crystal silicon
Substrate shape	Elliptical cylinder	Elliptical cylinder
Source to mirror centre	46.69 m	46.82 m
Mirror centre to focal point	0.31 m	0.18 m
Incidence angle at mirror centre	2.7 mrad	2.5 mrad
Reflection direction	Downward	Rightward
Substrate size (L × W × H)	150 × 30 × 50 mm	100 × 30 × 50 mm
Active optical surface (L × W)	140 × 8 mm	90 × 8 mm
Figure error (tangential)	<1.0 nm PV	<1.0 nm PV
Micro roughness	<0.15 nm RMS	<0.15 nm RMS
Sagittal radius	>10 km	>10 km
Reflective coating	Pt, 40–50 nm	Pt, 40–50 nm
Numerical aperture	6.1 × 10^−4^	6.2 × 10^−4^
Beam divergence	1.22 mrad	1.25 mrad
Geometrical demagnification	150.6	260.1
Beam acceptance aperture	378 µm	225 µm

**Table 5:** KB optics characteristics

	Photon energy		
	6 keV	10 keV	22 keV
Diffraction-limited focus (nm)	149	90	41
Focus simulation coherence mode (nm)	153	93	43
Focus simulation flux mode (nm)	188	113	52
SSA coherence mode (H × V) (µm)	21.5 × 12.8	12.9 × 7.7	5.9 × 3.5
SSA flux mode (H × V) (µm)	43 × 25.5	25.8 × 15.3	11.7 × 7.0
Depth of focus (µm)	550	330	150
